# Peripapillary choroidal neovascularization in pars planitis

**DOI:** 10.1186/1869-5760-3-13

**Published:** 2013-01-15

**Authors:** Sonia Mehta, Luxme Hariharan, Allen C Ho, John H Kempen

**Affiliations:** 1 Department of Ophthalmology, Emory University School of Medicine, Atlanta, GA, 30307, USA; 2 Ocular Inflammation Service, Department of Ophthalmology, Scheie Eye Institute, University of Pennsylvania, Philadelphia, PA, 19104, USA; 3 Retina Service, Wills Eye Institute, Philadelphia, PA, 19107, USA; 4 Department of Biostatistics & Epidemiology, Perelman School of Medicine, University of Pennsylvania, Philadelphia, PA, 19104, USA; 5 Center for Preventative Ophthalmology and Biostatistics, Perelman School of Medicine, University of Pennsylvania, 3535 Market St., Suite 700, Philadelphia, PA, 19104, USA

**Keywords:** Choroidal neovascularization, CNV, Intermediate uveitis, Pars planitis, Peripapillary

## Abstract

**Background:**

Choroidal neovascularization (CNV) is a rare complication of intermediate uveitis. Risk factors are not well-characterized. Here, we describe a case of peripapillary CNV in a patient with intermediate uveitis and explore the pathophysiology and treatment of this condition. This study is a case report and review of the literature.

**Results:**

A 15-year-old boy with intermediate uveitis - suppressed for the preceding year on immunosuppressive therapy and low-dose corticosteroids - and chronic disc swelling presented with unilateral metamorphopsia, peripapillary subretinal hemorrhage, and subretinal fluid. Fluorescein angiogram confirmed the presence of an active choroidal neovascular membrane. Treatment with intravitreal bevacizumab 1.25 mg every 4 weeks for 4 months resulted in resolution of subretinal fluid, subretinal hemorrhage, and regression of the CNV. The patient's intermediate uveitis remained inactive throughout this time.

**Conclusion:**

Review of the existing literature and pathophysiologic consideration suggests that chronic disc edema may be a risk factor for this condition. Peripapillary CNV in the context of intermediate uveitis appears to respond well to VEGF-inhibitor therapy.

## Findings

### Background

Choroidal neovascularization (CNV) occurs rarely in the context of intermediate uveitis [1]. Only three such cases have previously been reported in the English literature, two of which presented in a peripapillary location [1,2]. In the Systemic Immunosuppressive Therapy for Eye Diseases (SITE) Cohort Study, representing the experience of five US tertiary uveitis centers including ours, 0/1,978 patients with intermediate uveitis presented with peripapillary CNV (upper limit of a one-sided 97.5% confidence interval = 0.19%). Here, we report a case of peripapillary CNV in a patient with intermediate uveitis and explore the pathophysiology and outcome of this condition. The project was conducted in accordance with the principles of the Declaration of Helsinki, with the approval of the governing Institutional Review Board of the University of Pennsylvania.

### Case report

After completion of the SITE Cohort Study at our center, a 15-year-old male presented to our institution with blurry vision in the inferotemporal quadrant of the left eye for 1 day, which upon further questioning represented metamorphopsia. The patient denied redness, eye pain, light sensitivity, or floaters. The patient had longstanding intermediate uveitis and chronic disc swelling, which we observed to be suppressed for the past year. At the time of presentation with metamorphopsia, the patient was taking mycophenolate mofetil 1 g twice daily, methotrexate 25 mg once weekly, and folic acid 1 mg daily. He was otherwise healthy, and past medical history was unremarkable. Previous laboratory workup for conditions associated with intermediate uveitis was negative.

**Figure 1:**
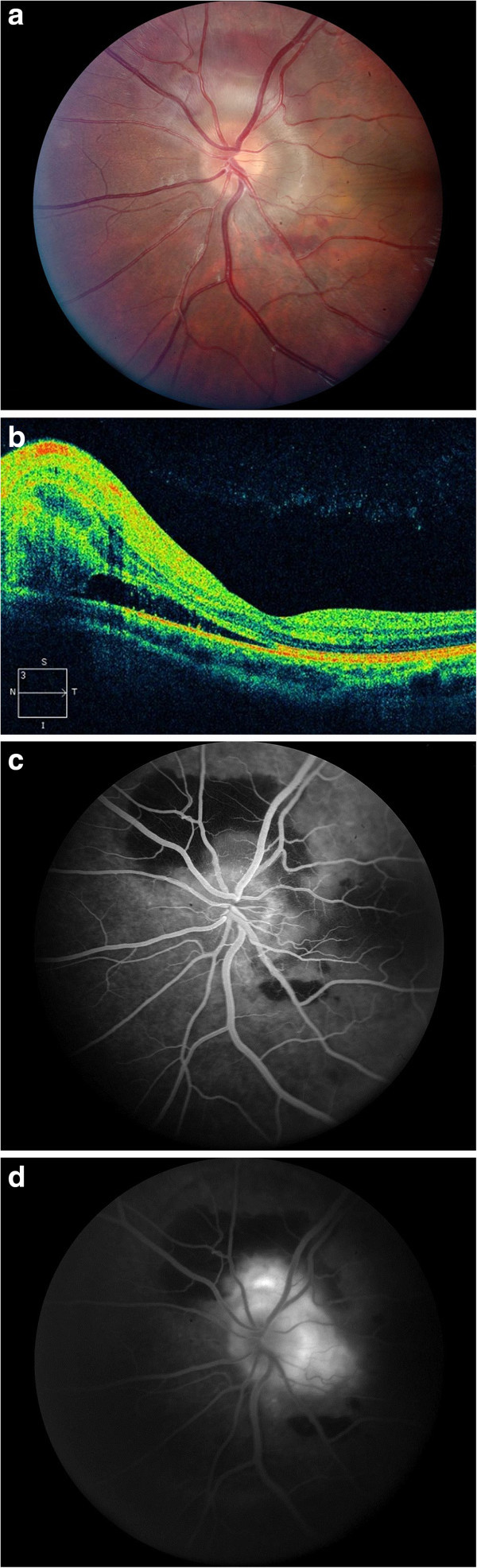
**Color fundus photograph, optical coherence tomography, and fluorescein angiogram at presentation.** (**a**) Color fundus photograph of the left eye at presentation demonstrating disc swelling with peripapillary subretinal hemorrhage and subretinal fluid. (**b**) Intraretinal and subretinal fluid temporal to the disc is evident on optical coherence tomography. (**c**) Early and (**d**) late fluorescein angiograms of the left eye reveals a choroidal neovascular membrane adjacent to the disc.

The patient's best corrected visual acuity was 20/20 in both eyes. Pupils, intraocular pressures, and color vision were normal. No afferent pupillary defect was present. Visual field was full in the right eye and showed an inferotemporal defect in the left eye. Slit lamp biomicroscopy was normal in both eyes showing no anterior or vitreous cells or flare. Fundus examination of the right eye showed stable chronic disc swelling but was otherwise unremarkable. In addition to disc swelling, the left eye fundus showed peripapillary subretinal hemorrhage and subretinal fluid extending into the macula (Figure 1a,b). Fluorescein angiogram showed an active choroidal neovascular membrane adjacent to the disc in the left eye (Figure 1c,d). There was no evidence of cystoid macular edema, periphlebitis, snowballs, or snowbanking in either eye on clinical exam, optical coherence tomography (OCT), and fluorescein angiogram. B-scan ultrasound showed bilateral disc elevation with fluid and positive 30° sign. Echography showed no evidence of optic disc drusen. MRI of the brain and orbits was normal; lumbar puncture revealed an opening pressure of 180 mm H_2_O at the upper limit of normal range.

**Figure 2:**
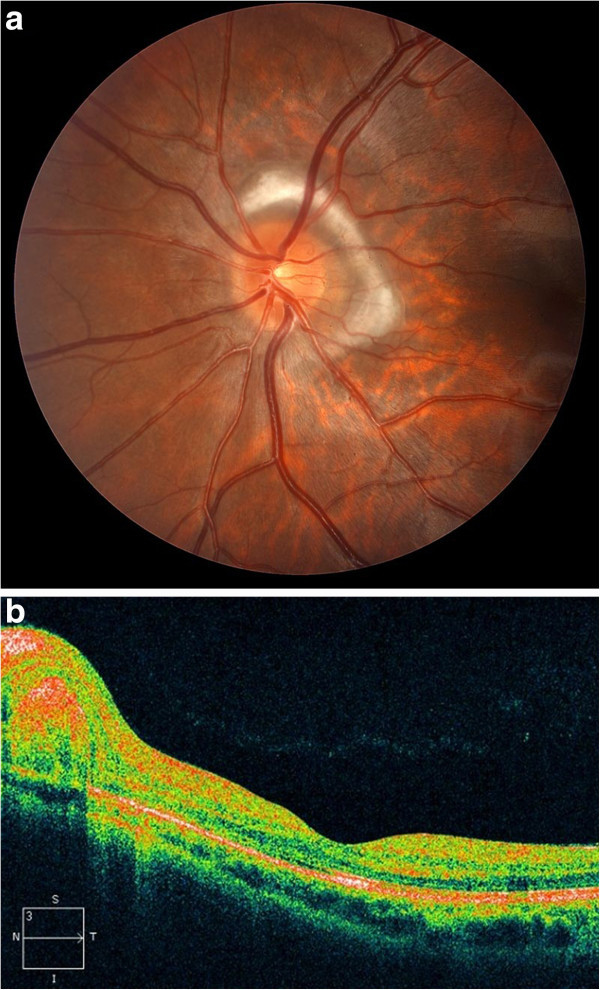
**Color fundus photograph and optical coherence tomography at follow-up.** (**a**) Color fundus photograph of the left eye 8 months after presentation showing resolution of the subretinal fluid and hemorrhage. (**b**) The subretinal fluid has resolved on optical coherence tomography.

The patient was treated with intravitreal bevacizumab 1.25 mg every 4 weeks for 4 months and retrobulbar triamcinolone acetonide 40 mg once at 6 weeks after presentation with the CNV. Acetazolamide 500 mg twice daily for several weeks after presentation did not decrease the disc swelling, at which point acetazolamide was discontinued. At 8 months follow-up, the patient's vision was stable and exam showed resolution of subretinal hemorrhage and fluid (Figure 2a,b) but continued traction lines in the area of the affected retina. Because the disc swelling gradually resolved over several months of follow-up, no further lumbar punctures were obtained.

Throughout this time, the patient's intermediate uveitis remained inactive. He was maintained on mycophenolate mofetil 1 g twice daily and methotrexate 25 mg once weekly. He was followed-up at our institution regularly over the next 3 years and did well. At last follow-up, the uveitis was inactive; no recurrence of CNV or disc swelling was noted throughout this period. His complaints of metamorphopsia gradually subsided, but he is still aware of metamorphopsia when he covers the right eye corresponding to the still-persistent traction lines in the nasal macula. It remains unclear whether the etiology of the disc swelling was from slowly resolving inflammatory disc edema, corticosteroid-induced intracranial hypertension, or both.

### Discussion

Most cases of CNV in uveitis occur in posterior uveitis or panuveitis. CNV in intermediate uveitis is rare with only three cases reported in the literature; two of the three cases were noted to occur adjacent to the disc (Table 1) [1,2]. Arkfeld and Brockhurst reported the first of these cases in a 29-year-old female with sarcoid-related bilateral intermediate uveitis [2]. The patient had disc swelling and active intermediate uveitis in the involved eye which was treated with oral prednisone. The disc swelling occurred in the involved eye 10 months prior to the onset of peripapillary CNV. She was treated with argon green laser photocoagulation and did well with CNV regression and significant visual improvement. Garcia et al. reported the second case of a 15-year-old female who presented with intermediate uveitis, vasculitis, papillitis, and peripapillary CNV [1]. The patient was treated with systemic corticosteroids and one dose of intravitreal bevacizumab 1.25 mg. On follow-up 7 months later, vision improved significantly, vasculitis resolved, and CNV regressed. Garcia et al. reported another case of a 27-year-old female with bilateral intermediate uveitis and chronic cystoid macular edema in the right eye [1]. Systemic workup for conditions associated with intermediate uveitis including sarcoidosis evaluation was negative. The patient was noncompliant with the treatment and presented 1 year after a flare-up with decreased vision and a subfoveal pigmented granuloma in the right eye which the authors described as regressed CNV.

**Table 1:** Literature summary of choroidal neovascularization in intermediate uveitis

	**Case**	**Age (year)/gender**	**Systemic workup**	**Location of CNV**	**Active intraocular inflammation at the time of CNV**	**Treatment**	**Outcome**	**Notes**
Arkfeld and Brockhurst [2]	1	29/female	Sarcoidosis	Peripapillary (temporal to disc)	No	Argon green laser photocoagulation	Regressed CNV	Chronic disc swelling present prior to CNV
Garcia et al. [1]	2	15/female	NR	Peripapillary-(temporal to disc)	Yes	Intravitreal bevacizumab × 1	Regressed CNV	Disc swelling present
Garcia et al. [1]	3	27/female	Idiopathic	Subfoveal	NR	None	Regressed CNV	Chronic cystoid macular edema present prior to CNV
Present report	4	15/male	Idiopathic	Peripapillary (superior and temporal to disc)	No	Intravitreal bevacizumab × 4	Regressed CNV	Chronic disc swelling present prior to CNV

*CNV*, choroidal neovascularization; *NR*, not reported.

Histopathologic studies have provided insight into the potential mechanisms in the development of peripapillary CNV [3]. Sarks provided clinicopathologic correlation of 21 eyes with peripapillary CNV from AMD [3]. Based on histopathologic evidence, Sarks hypothesized that peripapillary CNV originates either from the choroidal vessels passing around the termination of Bruch's membrane or from the choroidal vessels passing through defects in Bruch's membrane. In the above reported cases of peripapillary CNV in intermediate uveitis, it is interesting to note that chronic disc edema was present prior to the onset of all cases. Arkfeld and Brockhurst hypothesized that chronic disc swelling may increase the potential space between Bruch's membrane and swollen disc tissue facilitating ingrowth of choroidal vessels around the termination of Bruch's membrane [2]. Alternatively, chronic disc swelling may result in the deformation of the peripapillary Bruch's membrane leading to breaks in Bruch's membrane facilitating ingrowth of the choroidal neovascular tissue under the RPE [4]. Peripapillary CNV has also been observed in conditions causing chronic disc swelling such as idiopathic intracranial hypertension and hydrocephalus [5-8].

Peripapillary CNV is characterized clinically by the presence of a choroidal neovascular membrane adjacent to the disc producing subretinal hemorrhage, fluid, and/or exudate [4]. OCT characteristics suggestive of CNV include the presence of subretinal fluid, subretinal hemorrhage, subretinal hyperreflective lesion, and intraretinal fluid. On fluorescein angiogram, well-defined CNV appears as a lacy network of capillary plexuses with leakage as the angiogram progresses [9]. Occult CNV can appear as an area of stippled hyperfluorescence on angiogram. The choroidal neovascular membrane may be obscured by hemorrhage, pigment hyperplasia, fibrous tissue, or serous pigment epithelial detachment [9].

Peripapillary CNV has certain unique features compared to its macular counterpart. First, peripapillary CNV is associated not just with diseases affecting chorioretinal tissue adjacent to the disc but also with diseases affecting the optic nerve head. For example, peripapillary CNV can be seen in optic disc drusen, congenital disc anomalies, tilted disc, and disc swelling [4]. Second, peripapillary CNV may develop from the breakthrough of abnormal choroidal blood vessels through Bruch's membrane in addition to growth of vessels around the termination of Bruch's membrane [2-4]. Third, the occurrence of clinical symptoms prior to the extension of the lesion into the fovea may allow detection and treatment of these lesions with potentially more favorable visual outcomes compared to their foveal counterparts [4].

Several treatment options exist for peripapillary CNV in uveitis including vascular endothelial growth factor inhibitors, argon laser photocoagulation, and photodynamic therapy [10-13]. In the above cases, all patients underwent treatment with either argon laser photocoagulation or intravitreal bevacizumab (as used in our case) and did well with significant improvements in vision and CNV regression. For lesions adjacent to vital structures such as the optic disc, therapy with bevacizumab injection likely is safer than laser photocoagulation or photodynamic therapy and is reasonably cost-effective.

In conclusion, patients with intermediate uveitis and chronic disc swelling may be at risk for peripapillary CNV. Control of inflammation without the use of corticosteroids likely will improve disc swelling in most cases, even though resolution of disc swelling may be delayed in some instances, as in our case. Corticosteroid-induced or idiopathic intracranial hypertension should be suspected in cases with ongoing disc swelling without active inflammation. Chronic disc swelling should be resolved if possible to avoid complications. CNV has been reported to develop during times of quiescence or active inflammation [1,2]. Management with a vascular endothelial growth factor inhibitor likely is the treatment of choice, along with control of inflammation and control of any associated factors that may be present such as intracranial hypertension [13]. Based on the limited number of observations available, the prognosis of this condition with appropriate treatment appears favorable.

## Competing interests

JHK is a consultant at Lux Biosciences, Allergan, Alcon, Sanofi-Pasteur, and Xoma. All other authors have no competing interests.

## Authors’ contributions

SM and JHK designed and conducted the study. SM, LH, ACH, and JHK collected, managed, analyzed, and interpreted the data and prepared, reviewed, and approved the manuscript. All authors read and approved the final manuscript.

## References

[B1] Garcia CA Segundo Pde S Gardcia Filho CA Garcia AC Intermediate uveitis complicated by choroidal granuloma following subretinal neovascular membrane: case reports Arq Bras Oftalmol 2008 71 6 890 893 10.1590/S0004-27492008000600026 19169529

[B2] Arkfeld DF Brockhurst RJ Peripapillary subretinal neovascularization in peripheral uveitis Retina 1985 5 157 160 10.1097/00006982-198500530-00005 2416023

[B3] Sarks S New vessel formation between the retinal pigment epithelium in senile eyes Br J Ophthlamol 1973 57 951 965 10.1136/bjo.57.12.951 PMC1215217 4788954

[B4] Lopez PF Green WR Peripapillary subretinal neovascularization A review Retina 1992 12 2 147 171 1439246

[B5] Jamison RR Subretinal neovascularization and papilledema associated with pseudotumor cerebri Am J Ophthalmol 1981 91 312 317 563678 10.1016/s0002-9394(14)76669-3

[B6] Troost BT Sufit RL Grand MG Sudden monocular visual loss in pseudotumor cerebri Arch Neurol 1979 36 440 442 10.1001/archneur.1979.00500430070012 454249

[B7] Morse PH Leveille AS Antel JP Burch JV Bilateral juxtapapillary subretinal neovascularization associated with pseudotumor cerebri Am J Ophthalmol 1981 91 312 317 6163360 10.1016/0002-9394(81)90282-8

[B8] Nguyen C Borruat FX Bilateral peripapillary subretinal neovessel membrane associated with chronic papilledema: report of two cases Klin Monbl Augenheilkd 2005 222 3 275 278 10.1055/s-2005-857981 15786000

[B9] Gass JDM Gass JDM Diseases causing choroidal exudative and hemorrhagic localized (disciform) detachment of the retina and retinal pigment epithelium Stereoscopic atlas of macular diseases 1997 Mosby-Year Book, Inc., St. Louis 49 285

[B10] Mansour AM Arevalo F Ziemssen F Mehio-Sibai A Mackensen F Adan A Chan W Ness T Banker AS Dodwell D Tran THC Fardeau C Lehoang P Mahendradas P Berrocal M Tabbarah Z Hrisomalos N Hrisomalos F Al-Salem K Guthoff R Long term visual outcomes of intravitreal bevacizumab in inflammatory ocular neovascularization Am J Ophthalmol 2009 148 310 316 10.1016/j.ajo.2009.03.023 19427992

[B11] Arevalo JF Adan A Berrocal MH Espinoza JV Maia M Wu L Roca JA Quiroz-Mercado H Ruiz-Moreno JM Serrano MA Intravitreal bevacizumab for inflammatory choroidal neovascularization: results from the Pan-American Collaborative Retina Study Group at 24 months Retina 2011 31 353 363 10.1097/IAE.0b013e3181ed8cec 20890239

[B12] Lim JI Flaxel CJ LaBree L Photodynamic therapy for choroidal neovascularization secondary to inflammatory chorioretinal disease Ann Acad Med Singapore 2006 35 198 202 16625270

[B13] O'Toole L Tufail A Pavesio C Management of choroidal neovascularization in uveitis Int Ophthalmol Clin 2005 45 157 177 10.1097/01.iio.0000155902.49562.4a 15791164

